# Survival time to first antenatal care visit and its predictors among women in Ethiopia

**DOI:** 10.1371/journal.pone.0251322

**Published:** 2021-05-06

**Authors:** Abdu Seid, Mohammed Ahmed

**Affiliations:** 1 Department of Midwifery, College of Health Science, Woldia University, Woldia, Ethiopia; 2 Department of Public Health, College of Health Science, Woldia University, Woldia, Ethiopia; University of Mississippi Medical Center, UNITED STATES

## Abstract

**Background:**

First-trimester pregnancy stage is the fastest developmental period of the fetus, in which all organs become well developed and need special care. Yet, many women make their first antenatal visit with the pregnancy already compromised due to fetomaternal complications. This study aimed to fill this dearth using the 2016 national representative data set to augment early antenatal care visits in Ethiopia.

**Methods:**

A cross-sectional study design using the 2016 Ethiopia Demographic and Health Survey (EDHS) data set. Kaplan-Meir estimate was used to explain the median survival time of the timing of the first ANC visit. Multivariate Cox-proportional hazard regression analysis was performed to identify the factors related to the timing of the first ANC visit. Adjusted hazard ratios (AHR) with a 95% Confidence interval (CI) plus a p-value of < 0.05 were considered to declare a statistically significant association.

**Results:**

Data for 4666 study participants who had ANC follow-up history during pregnancy were included in the study and analyzed. The overall median survival time in this study was seven months. The timing of the first ANC visit was shorter by 2.5 times (AHR: 2.5; 95% CI: 2.34–3.68), 4.3 times (AHR: 4.3; 95% CI: 2.2–7.66), 4.8 times (AHR: 4.8, 95% CI: 4.56–10.8) among women who attended primary, secondary, and higher education as compared with non-educated one. Similarly, women who were residing in urban areas had 3.6 times (AHR: 3.6; 95% CI: 2.7–4.32) shorter timing of first ANC visit than rural residents. Furthermore, the timing of the first visit among the richest women was 3.2 times (AHR: 3.2; 95% CI: 2.5–9.65) shorter than the poorest women.

**Conclusion:**

The median survival time of the first ANC visit was seven months. The timing of the first ANC was longer among younger, poorer women, those who had no access to media, who considered distances as a big challenge to reach a health facility and, those with no education. Therefore, health care providers and community health workers should provide health education to create community awareness regarding the timing of the first ANC visit.

## Introduction

Despite pregnancy and childbirth being substantial events for women and their families, they are accompanied by a period of intensified vulnerability for both women and their unborn babies [1]. Even though improvement has been made in maternal health care utilization worldwide, maternal mortality and stillbirths remain the top global health challenge [2,3]. Globally, 830 women die every day, and more than 303,000 women die each year as a result of pregnancy and childbirth complications [4]. Of these, 99% of maternal deaths occur in emerging countries compared to 1% in industrialized nations [4]. In Ethiopia, the Maternal Mortality was 412 per 100,000 live births in 2016, one of the highest in the world [5].

According to the WHO’s focused antenatal care model recommendation, all pregnant mothers should begin ANC follow-up within the first trimester of pregnancy (within 12 weeks) [6]. In 2016, WHO revised its recommendations to a minimum of eight ANC contacts, with the first contact scheduled to take place in the first trimester (up to 12 weeks of gestation), two contacts scheduled in the second trimester (at 20 and 26 weeks of gestation) and five contacts scheduled in the third trimester (at 30, 34, 36, 38 and 40 weeks); this however, is not currently implemented in Ethiopia [7]. According to the Ethiopian Ministry of health recommendation, the timing of the first ANC visit is appreciable up to 16 weeks of gestation [5]. The 1^st^-trimester pregnancy stage is the fastest developmental period of the fetus, in which all organs become well developed and need special care [8,9]. Yet, many women make their first antenatal visit with the pregnancy already compromised due to fetomaternal complications [8,10].

According to studies done in Uganda, and Kenya the average timing of the first ANC visit was 7 and 5 months, respectively [11,12]. Likewise, another study conducted in Northwest Ethiopia reported that 52% of women booked their first ANC visit after 4 months of pregnancy [13].

Regarding factors about the timing of the first ANC visit, various studies showed that maternal age, educational status, wealth index, residence (rural or urban), husband education, pregnancy intention, women’s autonomy, distance to a health facility, pregnancy complication, knowledge on timely booking, having decision power to use antenatal care were significantly associated with timely commencement to antenatal care [13–17]. The late initiation of the first ANC visit could result in a missed chance of the prevention of complications, detection, and making an interventional plan for possible adverse pregnancy consequences [17]. Therefore, early initiation of the first ANC is a critical maternal health care service that helps in improving an extensive range of health outcomes for women and children through early detection of potential risk factors [18,19]. Even though timely initiation of ANC is essential, there is delayed initiation of the first ANC visit, and no emphasis is given to the determinants and the timing of the first ANC visit [20]. Even the national representative data from Ethiopia Demographic and Health Survey (EDHS) has limited data on these factors. Therefore, in order to fill the gap, this study assesses the predictors of survival time of first ANC attendance in Ethiopia using a recent version of the EDHS.

## Methods and materials

### Data source

The study used secondary data from the 2016 EDHS. A complete description of the design and methodology of 2016 is found elsewhere [5]. A nationally representative sample was obtained using a two-stage cluster sampling method. The first and second stages involved the selection of the clusters and households in each cluster, respectively. Further, stratification by rural-urban areas was taken into account. This study was based on data from the Woman’s Questionnaire, which was administered to all women aged 15–49 in the selected households.

#### Selection criteria

All women who had ANC attendance history during the current or last pregnancy within 5 years preceding the survey regardless of the outcome of pregnancy. The analytic sample for the current study comprised of 4666 women.

### Study variables

The time to first antenatal care visit is an outcome of interest which, is estimated in months. A woman who attended an ANC visit within the 1^st^ trimester was considered as an event whereas a woman who came to ANC visit within the 2^nd^ and 3^rd^ trimester was considered as a censored observation.

The independent variables were selected based on a literature review as being factors associated with the timing of the first ANC visit and includes women’s age, marital status, education level of the women, wealth index, residence (rural or urban), religion, media exposure, husband’s education, working status (occupation), distance to the health facility, health insurance and decision on health care service.

### Data processing and analysis

The data were analyzed using SPSS version 24. Descriptive statistics and weighted percentage were used to display the distribution of ANC booked women with respect to each trimester by their characteristics. The Kaplan-Meir estimate was used to explain the median survival time of the timing of the first ANC visit. Log-rank tests were used to select candidate variables for the multivariate model. Multivariate Cox-proportional hazard regression analysis was performed to identify the factors related to the timing of the first ANC visit. Adjusted hazard ratios (AHR) with a 95% Confidence interval (CI) plus a p-value of < 0.05 were considered to declare a statistically significant association. The model fitness was assessed by having -2loglikelihood chi-square value = 1493.53, *p* = 0.002. As recommended, sampling weights that accounted for complex survey design were incorporated in all analyses.

### Ethics approval and consent to participate

Ethical clearance for the study was not required since it is a secondary data analysis from EDHS 2016 database. The researchers received the survey data from USAID–DHS program and then the researchers of this study have maintained the confidentiality of the data. The consent was obtained from the study participants during EDHS data collection.

## Results

### Profile of the study participant related to the timing of antenatal care follow up

Data for 4666 study participants who had ANC follow-up history during pregnancy were included in the study and analyzed. Out of the women who had ANC follow-up, more than half (51.9%) were aged 25–34 years and started ANC visits in the first trimester of pregnancy. While 40.3% of them attended higher education and begin ANC visits within the first trimester of pregnancy. Likewise, the majority (90.9%) of women who were currently married or living with a man started their initial ANC visit within the first trimester of their pregnancy. Furthermore, out of women who had ANC follow-up, 56.9% of women residing in urban areas also began the first visit of ANC visit within three months of pregnancy. Similarly, 42.8% of orthodox religious followers began their first antenatal care follow-up in the first 3 months of pregnancy. Moreover, 44.8% of women within the richest wealth index category also started their first visit within the first trimester of pregnancy. On the contrary, 39.1% of women who lived far from health facilities did not start their first visit within the recommended time of initial visit.

The overall median survival time (MST) in this study was seven months which was computed from Kaplan-Meier estimates of the survivorship. The median survival time was slightly different in each subcategory of independent factors. For instance, the median survival time was 5 months for women with higher education levels compared to 9 months for women with no education. In summary women from the richest wealth index who had media access, an educated husband, and who were currently married to a man had shorter compared to their counterparts (Table 1).

**Table 1:** Percentage of study participants by their characteristics, median survival time, and log-rank test concerning the timing of the first ANC visit (n = 4666).

Variable	Categories	Timing of first ANC			MST in months	Log Rank test p-value
		1^st^ trimester	2^nd^ trimester	3^rd^ trimester		
Age	15–19	92(5.10%)	135(5.50%)	21(5.10%)	7	<0.001
	20–24	424(23.50%)	509(20.80%)	86(20.90%)	6	
	25–34	935(51.90%)	1251(51.00%)	196(47.60%)	7	
	35–49	350(34.40%)	558(22.70%)	109(26.50%)	7	
Educational status	No education	196(10.90%)	106(4.30%)	6(8.80%)	9	<0.001
	Primary	265(14.70%)	239(9.70%)	13(3.20%)	7	
	Secondary	614(34.10%)	810(33.00%)	109(26.50%)	7	
	Higher	726(40.30%)	1298(52.90%)	284(68.90%)	5	
Marital status	Never in union	20(1.10%)	20(0.80%)	3(0.70%)	8	0.02
	Currently in union/married	1638(90.90%)	2284(93.10%)	389(94.10%)	5	
	Formerly in union	143(7.90%)	149(6.10%)	20(4.90%)	7	
Residence	Urban	1024(56.90%)	1897(77.30%)	358(86.90%)	5	<0.001
	Rural	777(43.10%)	556(22.70%)	54(13.10%)	8	
Religion	Orthodox	770(42.80%)	921(37.50%)	140(34.00%)	5	0.01
	Catholic	14(0.80%)	14(0.60%)	0.00	6	
	Protestant	261(14.50%)	506(20.60%)	83(20.10%)	7	
	Muslim	743(41.30%)	996(40.60%)	182(44.20%)	7	
	Other	13(0.70%)	16(0.70%)	7(1.70%)	7	
Wealth index	Poorest	319(17.70%)	581(23.70%)	155(37.60%)	8	<0.001
	Poorer	223(12.40%)	456(18.60%)	76(18.40%)	7	
	Middle	233(12.90%)	382(15.60%)	78(18.90%)	7	
	Rich	219(12.20%)	391(15.90%)	58(14.10%)	7	
	Richest	807(44.80%)	643(26.20%)	45(10.90%)	4	
Occupation	Not Working	925(51.40%)	1293(52.70%)	242(58.70%)	6	<0.001
	Working	876(48.60%)	1160(47.30%)	170(41.30%)	7	
Husband education	No education	513(31.30%)	901(39.40%)	214(55.00%)	9	<0.001
	Primary	534(32.60%)	893(39.10%)	129(33.20%)	8	
	Secondary	309(18.90%)	292(12.80%)	32(8.20%)	7	
	Higher	282(17.20%)	198(8.70%)	14(3.60%)	5	
Media access	No	812(45.10%)	1429(58.30%)	299(72.60%)	7	<0.001
	Yes	989(54.90%)	1024(41.70%)	113(27.40%)	5	
The decision on health care service	Respondent alone	326(19.90%)	406(17.80%)	61(15.70%)	7	0.01
	Respondent and husband	1088(66.40%)	1482(64.9%)	235(60.40%)	7	
	Husband alone	224(13.70%)	395(17.30%)	93(23.90%)	7	
Health insurance	No	95(5.30%)	106(4.30%)	11(2.70%)	7	<0.001
	Yes	1706(94.70%)	2347(95.70%)	401(97.30%)	6	
Distance to a health facility	Big problem	704(39.10%)	1140(46.50%)	220(53.40%)	7	<0.001
	Not big problem	1097(60.90%)	1313(53.50%)	192(46.60%)	5	
Pregnancy intension	Then	1473(81.80%)	1937(79.00%)	321(77.90%)	6	0.03
	Later	259(14.40%)	361(14.70%)	67(16.30%)	7	
	No more	69(3.80%)	155(6.30%)	24(5.80%)	7	

**MST** = Mean Survival Time.

### Predictors of the timing of first antenatal care visit

All the variables were entered into multivariate Cox-proportional hazard regression analysis using the enter method. After adjusting for potential confounders by the regression, age, residence, religion, educational status of women and husband, occupation, wealth index, access to media, distance to the health facility, decision on health care and health insurance were significant predictors of timing of first ANC visit.

The timing of the first ANC visit was 1.20 (AHR: 1.20; 95% CI: 1.10–2.50), 1.80(AHR: 1.80; 95% CI 1.68–4.90) and, 1.90 times (AHR: 1.90; 95% CI: 1.13–2.38) shorter among women aged 20 to 24, 25 to 34, 35 to 49 compared to those aged 15 to 19 years, respectively. Likewise, the timing of the first ANC visit was shorter by 2.5 times (AHR: 2.50; 95% CI: 2.34–3.68), 4.30 times (AHR: 4.30; 95% CI: 2.20–7.66) and, 4.80 times (AHR: 4.8, 95% CI: 4.56–10.8) among women who attended primary, secondary, and higher education as compared to uneducated. Similarly, women who were residing in urban areas had 3.60 times (AHR: 3.60; 95% CI: 2.70–4.32) shorter timing of first ANC visit than rural residents. Furthermore, the timing of the first visit among the richest woman was 3.20 times (AHR: 3.20; 95% CI: 2.50–9.65) shorter than the poorest woman. Besides, the timing of the first ANC visit among Muslim religious followers was longer by 33% (AHR: 0.67; 95% CI: 0.62–0.73) than an orthodox religious follower. Women who did not have a big challenge to reach the health facility had 4.90 times (AHR: 4.90; 95% CI: 4.50–8.54) shorter timing of ANC visit than women who had a big challenge to arrive in a health facility (Table 2).

**Table 2:** Univariate and multivariate cox-regression analysis showing predictors of timing of first antenatal care visit in Ethiopia.

Variable	Categories	Unadjusted HR (CI: 95%)	Adjusted HR (CI: 95%)
Age	15–19	1	1
	20–24	1.90(1.60–2.70)**	1.20(1.10–2.50)*
	25–34	2.30(2.10–5.30)**	1.80(1.68–4.90)*
	35–49	1.90(1.11–1.46)**	1.90(1.13–2.38)**
Educational status	No education	1	1
	Primary	3.50(2.84–3.88)**	2.50(2.34–3.68)*
	Secondary	4.80(2.50–8.77)*	4.30(2.2–7.66)*
	Higher	5.3(4.96–13.8)**	4.80(4.56–10.80)**
Marital status	Never in union	1	1
	Currently married	1.30(0.89–1.79)	1.10(0.77–1.64)*
	Formerly in union	2.90(2.03–5.75)**	1.90(0.59–5.75)
Resident	Urban	5.10(4.36–6.22)**	3.60(2.70–4.32)**
	Rural	1	1
Religion	Orthodox	1	1
	Catholic	1.33(0.68–2.67)	1.10(0.33–9.59)
	Protestant	2.00(0.94–7.50)	1.61(0.78–6.62)
	Muslim	0.84(0.81–0.89)**	0.67(0.62–0.73)**
	Other	1.70(0.44–2.84)	1.30(0.21–1.95)
Wealth index	Poorest	1	1
	Poorer	2.30(1.87–9.52)*	1.90(0.69–6.35)
	Middle	2.90(2.23–6.28)*	2.30(1.55–4.73)*
	Rich	3.70(2.80–6.43)**	2.60(1.80–4.34)*
	Richest	4.87(3.68–10.56)**	3.20(2.50–9.65)**
Occupation	Not Working	1	1
	Working	2.50(2.22–4.82)*	1.50(1.35–3.67)*
Husband education	No education	1	1
	Primary	1.50(1.19–3.66)*	1.50(0.99–4.83)
	Secondary	2.10(1.90–4.52)**	1.20(1.19–5.30)
	Higher	3.80(2.53–8.94)**	2.40(1.86–5.22)**
Media access	No	1	1
	Yes	1.90(1.30–2.97)*	1.20(1.10–2.45)*
The decision on health care service	Respondent alone	2.60(1.87–4.86)**	0.88(0.39–9.87)
	Respondent and husband	3.80(3.45–9.47)**	1.20(1.10–6.60)*
	Husband/partner alone	1	1
Health insurance	No	1	1
	Yes	3.30(2.55–9.64)**	2.80(1.88–6.84)**
Distance to a health facility	Big problem	6.40(5.50–11.83)*	4.90(4.50–8.54)*
	Not a big problem	1	1
Pregnancy intension	Then	2.00(1.32–4.35)**	0.67(0.85–6.80)
	Later	1.70(0.67–8.70)	0.98(0.48–3.95)
	No more	1	1

*P-value <0.01

**P-value <0.001, HR = Hazard ratio.

## Discussion

WHO recommends that pregnant women should begin antenatal care in the first trimester of pregnancy [6]. This study revealed that the median survival time of pregnant women to initiate the first antenatal care visit was seven months. This finding was in line with a study done in Uganda [11], that reported the MST of the first ANC visit of7 months, but higher than studies done in Ghana, Nigeria, Kenya, Tanzania, and Pakistan [12,21–24], that reported a MST of less than 4 months. The variation might be due to socio-demographic and cultural barriers among Ethiopian women.

The study also showed that the timing of the first ANC visit was affected by age, residence, religion, educational status of women and husband, occupation, wealth index, access to media, distance to a health facility, decision on health care, and health insurance. In this study, women aged 20 to 24, 25 to 34, and 35 to 49 years have a shorter timing of antenatal care visits than women aged 15–19 years. This finding is supported by studies done by Nigeria [17] and Gondar [13]. The justification could be, the fact that awareness and level of health care decision-making increase with the age of the women [25]. Additionally, women younger than 20 years, could be delayed for the first ANC visit due to fear of the social consequence of teenage pregnancy, which include dismissal from school and stigma; adolescents are at high risk of undecided pregnancy disclosure [26]. Moreover, women who were residing in urban areas had a shorter timing of the first ANC visit than rural residents. The finding was consistent with studies done in Nigeria [17,27], Tanzania [23], and Uganda [11]. The consistency might be explained by the fact that pregnant women living in rural areas have no maternal health care services due to lack of availability, accessibility, and affordability as compared with urban residents [27]. Likewise, this study revealed that religion is one of the most important influencing factors for the timing of the first ANC services in which Muslim pregnant religious followers have a longer time for early initiation ANC visit as compared to the orthodox religious follower. This finding is also supported by a study done in Nigeria in which orthodox followers were 100% times more likely to initiate early ANC visits than Muslim women [17,28]. This is likely because most Christians go to spiritual places for care during pregnancy as most Christian organizations have mystical houses that enable to deliver a care for women particularly for their followers [29]. Likewise, a pregnant woman whose level of education was primary, secondary, and higher had a shorter timing of first ANC visit compared to those who had no education. This finding was similar to studies done in Ghana [24], Nigeria [27], Nepal [30], and Axum [31]. The plausible reason could be that educated women are better informed and, therefore, more aware of the importance of looking for ANC and other maternal health care services early compared with women with low or no formal education [32]. Similarly, this study revealed that women who had no big challenge to reach the health facility had a shorter timing of the first ANC visit than those who have a big challenge to arrive in the health facility. This finding is steady with studies done in Bahr Dar, Zambia, Cameron, and Tanzania [2,33–35]. This could be justified that the long-distance which takes more time to reach a health facility can influence patients not to seek appropriate maternal health care service at the health institution and can also be an enabling factor to why the women prefer not to have first ANC follow up at all [36]. Our finding also revealed that the poorest women have longer timing of the early initiation of the first ANC visits than their counterparts. This finding is supported by previous studies particularly done in Ethiopia, Pakistan, and Addis Ababa [14,22,37,38]. Financial barriers posed by high out-of-pocket costs for consultations may be an important determinant of low utilization of maternal health services, such as ANC, particularly in areas where scarcity is high and services are more costly [32]. Furthermore, this study found that women who were employed had a shorter time for early initiation of the first ANC visit than those women who were not employed, which is in line with studies done in Addis Ababa [37,39]. Economically empowered and educated women may put more emphasis on maternal and child health care decision which could help to initiate early ANC utilization [40].

Women’s participation in the decision-making process is crucial to increase utilization of maternal health care services which helps to reduce maternal mortality. As perceived in this study, mothers who decided jointly with their husband had a shorter time for early initiation of first ANC visit compared to those respondents whose husband made the decision alone, which is consistent with a previous study done here in Ethiopia [14]. This could be due to the fact taking part in the decision-making process helps the women to get support from a husband that in turn increases early initiations of the first ANC visit and utilization of services [13]. Besides, women who had health insurance have shorter timing of first antenatal visit, which is in line with a study done in low and middle-income countries in which the proportion of women reporting the timing of first ANC visit was shorter among insured than uninsured women [41]. Similarly, this finding is also supported by studies which were done in Gabon [42] and Ghana [43–45] and, Tanzanian [46]. The justification of this finding could be that the implementation of health insurance is one of the possible health financing mechanisms to increase maternal health care service utilization including early initiation of ANC by reducing out-of-pocket costs [47,48]. Furthermore, mass media may also play an important role in increasing the use of antenatal care and other maternal health services. In this study, women who had access to media have a shorter timing of the first antenatal care visit than their counterparts which was in line with previous studies done in Pakistan, Nigeria, and Nepal [22,27,49]. Utilizing mass media to disseminate ANC message may be an effective approach to improve the uptake of early initiation of the first ANC visit [50].

## Conclusion

In this study, the median survival time of the first ANC visit was seven months which is highly delayed as compared to WHO recommendation of ANC visit. Furthermore, women in younger age group classified as poorer in wealth quintile, had no access to media, lived far from the health facility, with no education, living in rural areas, were Muslim religious follower, had no health insurance, and had decided alone on health care decision have longer timing of the first ANC visit. Therefore, all responsible bodies should take a conclusive and proper intervention to prevent adverse fetomaternal outcomes due to the delayed initiation of ANC visits. Health care providers and community health workers should provide health education to create community awareness regarding the timing of the first ANC visit. Finally, the Ethiopian Ministry of Health and Regional Health Bureau have to design a strategy to increase the number of women in the utilization of early ANC follow up and as well they should build health care facilities closer to people especially for women residing in a rural area.

## Abbreviations

AHR: adjusted hazard ratio
ANC: antenatal care
CI: confidence interval
EDHS: Ethiopia Demographic and Health Survey
SPSS: Statistical Package for Social Science
WHO: World Health Organization
